# Overview of Stigma against Psychiatric Illnesses and Advancements of Anti-Stigma Activities in Six Asian Societies

**DOI:** 10.3390/ijerph17010280

**Published:** 2019-12-31

**Authors:** Zhisong Zhang, Kaising Sun, Chonnakarn Jatchavala, John Koh, Yimian Chia, Jessica Bose, Zhimeng Li, Wanqiu Tan, Sizhe Wang, Wenjing Chu, Jiayun Wang, Bach Tran, Roger Ho

**Affiliations:** 1 Faculty of Education, Huaibei Normal University, Huaibei 235000, China; rsczzs@chnu.edu.cn (Z.Z.); lizhimeng1978@163.com (Z.L.); chuchu0117@outlook.com (W.C.); pcmrhcm@nus.edu.sg (R.H.); 2 Institute for Health Innovation and Technology (iHealthtech), National University of Singapore, Singapore 119077, Singapore; cjytwq@163.com; 3 Department of Family Medicine and Primary Care, The University of Hong Kong, Hong Kong, China; kssun2@hku.hk; 4 Department of Psychiatry, Faculty of Medicine, Prince of Songkla University, Songkhla 90110, Thailand; jchonnak@gmail.com; 5 Faculty of Medicine, University of New South Wales, Sydney, NSW 2052, Australia; mingyanjohn.koh@student.unsw.edu.au; 6 Department of Psychological Medicine, Yong Loo Lin School of Medicine, National University of Singapore, Singapore 119077, Singapore; chiayimian96@u.nus.edu (Y.C.); e0035467@u.nus.edu (J.B.); 7 The China-Singapore (Chongqing) Demonstration Initiative on Strategic Connectivity Think Tank, Chongqing 400043, China; 8 School of Mathematics, Jilin University, Changchun 2699, China; wangsz812@163.com; 9 Institute for Preventive Medicine and Public Health, Hanoi Medical University, Hanoi 100000, Vietnam; bach.ipmph@gmail.com; 10 Johns Hopkins Bloomberg School of Public Health, Baltimore, MD 21205, USA; 11 Centre of Excellence in Behavioural Medicine, Nguyen Tat Thanh University, Ho Chi Minh City 70000, Vietnam

**Keywords:** Asia, depression, bipolar disorder, schizophrenia, stigma

## Abstract

*Background*: In psychiatry, stigma is an attitude of disapproval towards people with mental illnesses. Psychiatric disorders are common in Asia but some Asians receive inadequate treatment. Previous review found that Asians with mental illness were perceived to be dangerous and aggressive. There is a need for renewed efforts to understand stigma and strategies which can effectively reduce stigma in specific Asian societies. The objective of this systematic review was to provide an up-to-date overview of existing research and status on stigma experienced by psychiatric patients and anti-stigma campaigns in China, Hong Kong, Japan, Singapore, Korea, and Thailand. *Methods*: A systematic literature search was conducted in the following databases, including PubMed, PsycINFO, Embase, Web of Science, and local databases. Studies published in English and the official language of included countries/territories were considered for inclusion in the systematic review. Any article on stigma related to any form of psychiatric illness in the six Asian societies was included. *Results*: One hundred and twenty-three articles were included for this systematic review. This review has six major findings. Firstly, Asians with mental illnesses were considered as dangerous and aggressive, especially patients suffering from schizophrenia and bipolar disorder; second, psychiatric illnesses in Asian societies were less socially-acceptable and were viewed as being personal weaknesses; third, stigma experienced by family members was pervasive and this is known as family stigma; fourth, this systemic review reported more initiatives to handle stigma in Asian societies than a decade ago; fifth, there have been initiatives to treat psychiatric patients in the community; and sixth, the role of supernatural and religious approaches to psychiatric illness was not prevailing. *Conclusion*: This systematic review provides an overview of the available scientific evidence that points to areas of needed intervention to reduce and ultimately eliminate inequities in mental health in Asia.

## 1. Introduction

Stigma is an attitude of disapproval towards a specific group of people with different characteristics [1]. Discrimination occurs when a group of people with stigmatizing attitudes deny another group of people of their rights by exclusion and marginalization [2]. In psychiatry, stigma can be defined as a distinguishing demarcation between the people with and without psychiatric illnesses, attributing negative characteristics of psychiatric illnesses to this person [3]. Stigma can lead to negative discrimination, low self-esteem [4], psychological burden, and ultimately interfere with psychiatric services [5]. Stigma can lead to a negative impact on adherence and attitude towards psychiatric treatment [6]. Discrimination against people diagnosed with psychiatric illnesses may lead to delays and avoidance of psychiatric treatments [7]. Stigma and discrimination are the most significant challenges that people living with chronic diseases face, leading to a negative quality of life [8,9].

Globally, more than 70% of young people and adults with psychiatric illnesses do not receive any psychiatric treatment [10]. In particular, Asia ranked second in terms of the prevalence of common psychiatric disorders (e.g., depression) and these patients may not receive adequate psychiatric treatment [11]. Asia is a continent with cultural and economic diversity which affects the care [12] and rehabilitation [13] for psychiatric patients. In 2007, Lauber and Rössler reviewed about stigma towards people with mental illness in developing countries in Asia [14]. The findings are summarized as follows: (1) Asians with mental illness were considered to be dangerous and aggressive; (2) lack of personal and financial resources to handle stigma; (3) mental health professionals mainly worked in urban areas; (4) somatic symptoms were more socially acceptable than psychiatric symptoms; (5) stigma experienced by family members was pervasive; and (6) the role of supernatural and religious approaches to psychiatric illness was prevailing. After a decade, there is a need for renewed efforts to understand stigma and strategies which can effectively reduce stigma in specific Asian societies.

Therefore, we proposed a systematic review to summarize the available literature on stigma related to psychiatric illnesses in six Asian societies, including China, Hong Kong, Japan, Korea, Singapore, and Thailand. First, we discuss the current stigma situation in these Asian countries. Next, we present an overview of anti-stigma activities in these Asian countries. Finally, we present our views and recommend future research directions.

## 2. Methods

### 2.1. Objectives

The objective of this systematic review was to provide an overview of existing research and status on stigma experienced by psychiatric patients and anti-stigma campaigns in China, Hong Kong, Japan, Singapore, Korea, and Thailand.

### 2.2. Inclusion and Exclusion Criteria

**Figure 1:**
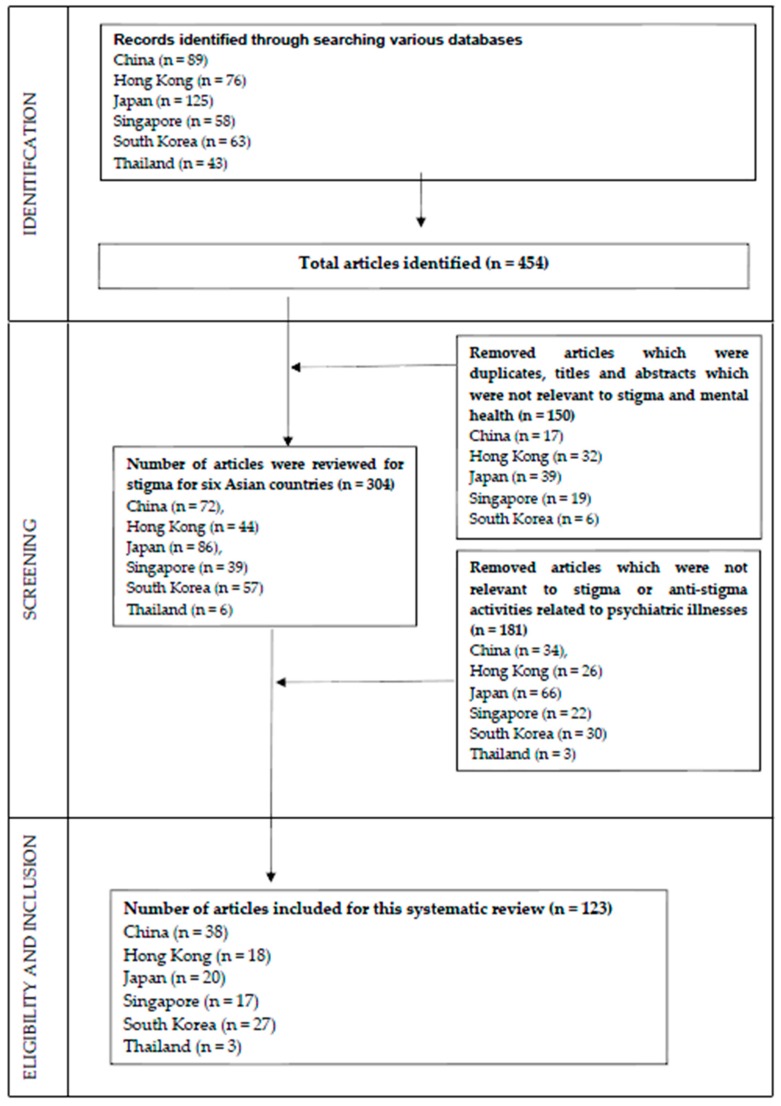
Full screening process in accordance to PRISMA guidelines.

Articles published in English and the official language of included countries/territories were considered for inclusion in the systematic review. Any article on stigma related to any form of psychiatric illness in China, Hong Kong, Japan, Korea, Singapore, and Thailand was included in this systematic review. The selection process is based on Preferred Reporting Items for Systematic Reviews and Meta-Analyses (PRISMA) (see Figure 1). There was no exclusion of article based on the type of article (e.g., quantitative or qualitative study), methods to measure stigma, the background of participants who reported stigma and how the participants were identified. We only excluded papers that were not conducted in China, Hong Kong, Japan, Korea, Singapore, and Thailand.

### 2.3. Search Strategy

#### 2.3.1. Pre-Identification Stage

We performed a literature review in the following databases, including PubMed, PsycINFO, Embase, Web of Science, as well as local databases. We extended our search back to the inception of databases. Keywords of ‘mental’, ‘psychiatric, ‘stigma’, ‘discrimination’, and the name of the respective country were used. An analysis of text words contained in the title and abstract of retrieved papers was conducted.

#### 2.3.2. Identification Stage

Articles related to stigma in six Asian countries/territories were identified and screened. We removed articles that were duplicates and not relevant to stigma and psychiatric illnesses. We further examined articles that were relevant to stigma and anti-stigma activities related to specific psychiatric illnesses in six Asian countries/territories.

## 3. Results of Systematic Review

The selection process of articles is illustrated in Figure 1. The search strategies identified 494 articles from database searching and hand-search of relevant peer-reviewed journals. From this original hit, 150 articles which were duplicates, and titles and abstracts which were not relevant to stigma and mental health were removed. Three hundred and four articles were reviewed for stigma for six Asian countries. One hundred and eighty-one articles which were not relevant to stigma or anti-stigma activities related to psychiatric illnesses were removed. Finally, 123 articles were included for this systematic review.

### 3.1. Stigma Related to Psychiatric Illnesses and Advancement of Anti-Stigma Activities in China

China is one of the largest countries in Asia, and its population had reached 1.395 billion in 2018. There are about 130 million Chinese suffering from mild to severe psychiatric disorders [15]. Around 7.8 million Chinese suffer from schizophrenia, accounting for 9.2 percent of the total number of Chinese with disabilities. There are 100,000 new cases of schizophrenia per year [16,17]. Several studies have shown widespread discrimination against Chinese with psychiatric illnesses. A study found that 67.6% of participants agreed that society discriminates against people with mental disorders more seriously than other disabled people [18]. Only 31.40% of the residents held a positive attitude towards psychiatric illnesses [19,20]. Another study reported widespread discrimination against people with mental illnesses by family members, nurses, and students [21], although such discrimination was less than that imposed by the general public [22]. Furthermore, rural residents are more discriminating than urban residents, which may be related to factors such as understanding of mental illnesses [23].

From patients’ perspectives, 80% of psychiatric patients experienced discrimination [24,25]; 69.0% of psychiatric patients believed that illness would affect their job application [26,27] and 42% of schizophrenia patients believed that they were treated unfairly by work colleagues, neighbors, and family members [28,29,30]. There was an inverse relationship between stigma and duration of psychiatric illness with patients suffering from less than five years of illness perceiving more stigma than those with longer than five years of illness [31]. Psychiatric inpatients with higher levels of perceived stigma reported lower levels of quality of life [32].

Patients’ families are caregivers of psychiatric patients, but are also discriminated against by others; they sometimes even discriminate against themselves. The incidence of stigma experienced by Chinese caregivers of schizophrenia patients was 78.3% [33]. Around 56% of family members kept the psychiatric diagnosis of family members secret to avoid discrimination, and 75% believed that discrimination would cause stress on family members [34]. Discrimination was found to reduce the social status and self-esteem of patients and their families [35]. As a result, around 26% of family members alienated their relatives who are psychiatric patients [26].

Health care workers have close contact with psychiatric patients, and their attitude directly affects treatment adherence. Around 35.2% of Chinese mental health professionals admitted a discriminating attitude against psychiatric patients [36]. Studies have shown differences in the levels of discrimination against people with psychiatric disorders among psychiatric nurses as compared to other health care workers. Li et al. (2012) reported that nurses working in psychiatric hospitals demonstrated more discriminatory attitudes towards psychiatric patients than nurses working in general hospitals [37]. In contrast, Zeng et al. (2017) found that non-psychiatric nurses had a higher degree of discrimination against patients with mental illnesses than psychiatric nurses [38]. In addition, nurses working for psychiatric hospitals were more accepting towards psychiatric risk assessment than nurses in general hospitals [39].

Recent studies show that university students from different disciplines show different attitudes toward psychiatric patients. Medical students showed less discrimination against psychiatric patients than nursing students [40,41]. The degree of discrimination was higher among senior nursing students than junior nursing students [42]. Nevertheless, nursing students showed less discrimination against psychiatric patients than students from non-medical disciplines [43,44]. In general, community residents without a university degree demonstrated higher levels of discrimination than university students from medical and non-medical disciplines [45].

Media coverage is an important factor influencing people’s attitudes towards people with mental illnesses in China. Zeng et al. (2009) studied all media reports involving psychiatric patients in China from 2005 to 2006, which showed that most reports involved negative themes, and few reports were positive about fighting mental illnesses. Most reports do not intentionally discriminate against people with mental illnesses, but the descriptions of their behavior create a negative image of this group. Moreover, journalists have little knowledge of psychiatric disorders, which also affects the accuracy and fairness of their report. Around 68.6% of journalists reported that they only had superficial knowledge about mental illnesses; 11.4% of journalists had a systematic understanding of mental illnesses, and 20.0% of journalists had special knowledge of mental illnesses [46]. In another study, content analysis was performed on 640 reports from People’s Daily, an official and widely-read newspaper in China, on psychiatric patients from 1 May 2013 to 1 May 2017 [19]. This study found that 81.9% of the reports held negative attitudes. Most reports reported abnormal or violent behavior of psychiatric patients, and threats posed by them to other people and society. Only 3.9% of the stories were positive, which made it difficult for readers to see the positive side of psychiatric disorders. Furthermore, reports often refer to the group as “mental patients” rather than referring to specific psychiatric illness and circumstances of a patient (e.g., non-adherence to treatment). It might mislead readers to believe that most psychiatric patients are dangerous. As a result, there were calls for the media to adopt a neutral stance when reporting news related to psychiatric patients [47].

The Chinese government has recognized that discrimination against people with mental illnesses is a serious social problem and not in line with the policy on a harmonious society. The National Mental Health Work Plan (2015–2020) requires the media to extensively publicize correct information about mental health, including that psychiatric disorders are treatable; early interventions exist for psychiatric disorders, and psychiatric disorders are similar to chronic medical diseases. The media is required to standardize the reports on incidents related to psychiatric patients. Chinese citizens are calling for more respect and care for people with mental illnesses through national legislation and public education to reduce discrimination and stigma. Examples include Article 4 of the Mental Health Law of the People’s Republic of China (revised in 2018), which stipulates the legitimate rights and interests of psychiatric patients. The personal information of psychiatric patients should be kept confidential. Article 5 stipulates that no organization or individual can discriminate, insult, or maltreat psychiatric patients or illegally restrict their freedom. Media reports, literature, and artistic works shall not contain any content that discriminates psychiatric patients. Article 22 stipulates that the state should support public welfare policy for psychiatric patients. In addition to the above legislation, some academics have proposed anti-discrimination law which can effectively protect the legitimate rights and interests of psychiatric patients in China [48].

Previous research found that if citizens had more knowledge about mental illnesses, the degree of discrimination against psychiatric patients would reduce [38,49,50,51,52]. Calls were made on the society to pay more attention to mental health, disseminate correct knowledge on mental illnesses, help people to treat psychiatric patients rationally and objectively. Health authorities can invite the public to visit psychiatric hospitals and participate in voluntary work. This approach will provide opportunities for the public to have close contact with psychiatric patients, reducing their fear but enhancing their understanding, which will reduce ultimately discrimination.

### 3.2. Stigma Related to Psychiatric Illnesses and Advancement of Anti-Stigma Activities in Hong Kong

Hong Kong has been a special administrative region of the People’s Republic of China since 1997. Before 1997, it was a British colony, but the majority of residents of Hong Kong are Chinese. Comparative studies showed that the Chinese had higher stigma towards mental illnesses than the Westerners, reflected by indicators such as social distance and negative stereotypes [53,54]. A comparative survey found that the Chinese were more likely than the British to perceive that people with schizophrenia are dangerous, uncontrollable, and act abnormally [53]. A qualitative study in Hong Kong [55] found that the general public held negative stereotypes on the potentially violent and annoying behaviors of patients with schizophrenia, mania, and bipolar disorders. However, stigmatizing opinions did not generalize across different psychiatric illnesses. Patients with anxiety disorders and depression were better accepted than those with psychosis. This pattern was also observed in a survey comparing primary care physicians’ attitudes towards schizophrenia and depression [56].

In the Chinese context, the behaviors of psychiatric patients are not solely their personal issues but also relate to the public interest [55]. There are views that the stereotypes of dangerousness and unpredictability towards psychiatric patients violate cultural norms of restrained and moderate behavior for social order, which is emphasized by Confucianism [54,55,57,58]. The deep concern about shame and loss of “face” in Chinese culture may also intensify the stigma on psychiatric patients [59]. While these arguments fit into the context for psychotic disorders, the stigma for depression and anxiety disorders relates more to weakness in personality and having bad thoughts [59,60,61]. Regardless of the type of stigma, it lowers patients’ willingness to seek help and affects their social life and employment opportunities [62]. Difficulties in job-seeking are common among psychotic patients. A recent study found that 71% of the patients with schizophrenia living in the community were unemployed [63]. The number of relapses during the first three years and low level of education are predictors for unemployment among patients suffering from first-episode schizophrenia-spectrum disorders [64]. Apart from public stigma, patients often have self-stigma which lowers their self-esteem. A study on patients with schizophrenia found that self-stigma was associated with weaker social support, interaction, and functioning, which in turn affected their symptoms and recovery [65].

For anti-stigma interventions, schizophrenia and depression are the most common psychiatric conditions to be targeted due to the high public stigma for the former and high prevalence for the latter. A recent systematic review of studies on the general public suggested a small effect of the interventions on reducing negative stereotypes and improving mental health literacy [66]. Lectures, role-plays, videos, and educational materials were used to improve mental health literacy and challenge myths about mental illnesses. Reducing perceived stigma and enhancing coping with stigma lead to improvements in quality of life, social support, and functioning [67]. Psychoeducation and cognitive behavior therapy are common approaches to reduce stigma. Family members and caregivers play an important role in anti-stigma interventions.

Apart from intervention studies by researchers, there are strategies used by the medical professions and policymakers to reduce public stigma on mental illnesses. In Hong Kong, the Chinese translation of psychosis was officially renamed from ‘jingshen-fen-lie’ (meaning a split in mind) to ‘si-jue-shi-tiao’ (meaning incoordination in thoughts and perceptions) in 2001. It aimed to replace the negative meaning of the old term ‘mental split-mind disorder’ with a more objective meaning ‘dysregulation of thoughts and perception’ [68]. The term ‘psychiatry’ is often avoided in private psychiatric clinic names. Instead, other terms in Chinese such as ‘psychosomatics’, ‘mind’, or ‘heart’, ‘peace’, ‘spirit’, and ‘joy” are commonly used [69]. Experience from Hong Kong show some effects of renaming of psychosis on public stigma [68,70]. Nevertheless, some journalists still reserve the old term for reporting negative news with dangerous wordings [68]. More research is needed to explore the effect of terminology on reducing public stigma on mental illnesses.

### 3.3. Stigma Related to Psychiatric Illnesses and Advancement of Anti-Stigma Activities in Japan

In recent decades, Japan’s mental health care system has made great efforts to improve its services available to the public. Despite this, most individuals who meet the Diagnostic and Statistical Manual criteria for psychiatric disorders report not having sought treatment [71]. The lack of help-seeking behavior has been attributed to the stigma associated with mental illness in Japan. One psychiatric illness in particular that has been of much concern is schizophrenia. In 1937, Seishin-Bunretsu-Byo (‘mind splitting disease’) was approved as the official translation for schizophrenia in Japan. In 1993, a proposal for the renaming of the condition was made because Seishin-Bunretsu-Byo ‘was humiliating’ [72]. In 2002, Togo-Shitcho-Sho (‘integration disorder’) was officially announced as the new term for schizophrenia [73]. The intention of dispelling the social disadvantage associated with the diagnosis was met with some success. The Japanese Society for Psychiatry and Neurology, mental health professionals and persons with schizophrenia and their families accepted the new name. As a result, the rate of informing patients of their diagnosis had risen from 37% in 2002 to 70% as of 2004; the rate of inpatients reporting their diagnosis has also increased from 16% in 1977 to 55% in 2014 [74]. The general public acquire more knowledge of and hold fewer negative stereotypes for the new name of schizophrenia [75]. In line with the efforts to diminish the stigma surrounding mental illness, including schizophrenia, goals of treatment have expanded beyond alleviating psychiatric symptoms to the successful assimilation into a community where one can achieve social well-being [76].

However, despite some endorsement of the reconceptualization of mental illness in Japan, it is evident that deep-rooted stigma toward the mentally ill still exists. Even though the opinion from the general public is that mental illness can be treated [77], stigma toward psychiatric patients—and those with schizophrenia in particular—is still relatively strong [78,79,80]. These lingering negative attitudes can be seen in the way Japanese living in communities often oppose to the construction of public facilities for the mentally ill [76]. In addition to the former derogatory term for schizophrenia, many years of newspaper coverage of the condition as dangerous shaped the beliefs and attitudes of the public [81]. Since the renaming, there have been less stigmatizing articles regarding schizophrenia. However, this progress was ironically met with an increase in stigmatizing articles about bipolar disorder [81]. Such stigma toward mental illness often leads to difficulty forming trusting relationships and interferes with medical treatment by preventing help-seeking behaviors [76,80,82] The consequences of individuals resorting to self-management of their illness can be dire, especially for those who struggle with depression. The stigma surrounding depression in Japan, while relatively less than that of schizophrenia is still pronounced among the general population [75]. A typical Japanese belief is that depression is caused by a weak personality [83]. These stigmatized individuals tend to form the belief that suicide could be the ultimate solution instead of seeking help [84,85]. The annual number of suicides in Japan exceeded 30,000 between 1998 and 2011, calling for an urgent need to establish effective suicide prevention strategies [82]. Increasing research has gone into programs targeted at reducing stigma surrounding mental illness, with findings that educational programs [86,87] and social contact [88] are effective interventions with social contact appearing to have more long-term benefits. Moving forward, there is a need to make psychiatric services more easily accessible to adolescents [89] and to address problems regarding institutionalism and societal homogeneity in Japan—which may account for the stronger stigmatizing attitudes as compared to countries like Australia and the US [90].

### 3.4. Stigma Related to Psychiatric Illnesses and Advancement of Anti-Stigma Activities in Singapore

Singapore is a metropolitan city-state in South East Asia, with a total population of 5.64 million as of 2018. The country is notably multi-ethnic with Chinese (76.1%), Malay (15.0%), and Indian (7.5%) [91]. The 2010 Singapore Mental Health Study (SMHS) found that 12.0% of respondents had at least one affective, anxiety, or alcohol-use disorder in their lifetimes. The lifetime prevalence of major depressive disorder was 5.8% [92]. Disorder severity negatively predicted mental health component of health-related quality of life (HRQOL) for Singaporeans suffering from depression [9]. Among Singaporean psychiatric outpatients with depression and anxiety disorders, 43.6% experienced moderate to high levels of self-stigma [93]. For patients with schizophrenia, the number of hospitalizations negatively predicted the mental health component of HRQOL [94]. A significant negative relationship between QOL, self-esteem and general functioning and self-stigma was observed [95].

There is stigma associated with seeking treatment for mental health problems. Non-psychotic psychiatric patients (e.g., mood disorders) felt more stigmatized for visiting a state mental hospital as compared to a general hospital for outpatient treatment. They reported difficulty in finding employment (59.2%) and rejection by insurance companies (27.7%). Chee et al. (2005) reported an “institutional” effect whereby patients experienced additional stigma associated with a particular treatment setting and resulting in community sanction [96]. In another study, around 38.3% of respondents believed that people with mental illnesses were dangerous, and 49.6% felt that the public needed to be protected from psychiatric patients [97]. A negative attitude towards people with mental illness correlated with lower educational background and greater age [97]. Individuals with higher stigma scores, particularly those in aged 65–69 years, also indicated that they found it difficult to talk to psychiatric patients and felt that patients had to blame themselves for their conditions [97]. A vignette-based study assessing the attitudes of Singaporeans found that 89.4% of respondents agreed that individuals with depression could get better if they were adherent to treatment [98]. Around 50.8% of respondents indicated that mental illness is a sign of personal weakness [98].

Singapore is a multi-cultural city-state with three major ethnicities. The Malay Singaporeans viewed mental illnesses with the highest level of weak-not-sick scores but reported the lowest social distance score to people who have mental illnesses [98]. Singaporeans of Indian ethnicity rated mental illnesses with the highest dangerous–undesirable scores and reported middle-range social distance scores to people with mental illnesses [98]. The Chinese Singaporeans reported the highest social distance scores to people with mental illnesses [98]. Similarly, another study assessing adolescent attitudes suggests that young Chinese Singaporeans display a higher sense of physical threat and lower social tolerance towards people who are mentally ill than youths of other ethnicities [99].

For psychotic illness, Singaporean with first-episode psychosis reported a high level of stigma after 1-year of follow-up. Around 71.3% of participants experienced at least one episode of discrimination. The most common experiences of discrimination included being shunned by people who were aware of their mental health issues (28.7%), difficulty in making and keeping friends (24.7%), discrimination by family members (22.9%), and discrimination by mental health staff (22.8%) [100].

Anti-stigma activities in Singapore are conducted by the Ministry of Health (MOH) and non-profit organizations. In 2006, the MOH launched the National Mental Health Blueprint (NMHB) to promote primary prevention, improving the coordination of psychiatric services, developing mental health professionals, enhancing mental health monitoring and the quality of psychiatric services [101]. The NMHB signified the beginning of a policy shift from an institution-based model of care to a community-based approach [102]. Thus, the destigmatization of mental illness in the community is a key focus of the Blueprint. The Health Promotion Board (HPB) is the main driver for mental health promotion. Through improving understanding of mental illnesses and their symptoms, the HPB aims to encourage people to seek help, as well as reducing stigma and discrimination in the community. The HPB has school-based program such as educating students on mental health. Mental Health First Aid courses are organized for stakeholders and teachers to improve their mental health literacy. In the workplace, the HPB runs seminars for human resource professionals to raise awareness and participates in the employers-led Mental Health Alliance. Public education and community outreach events aimed at fighting stigma are organized collaboratively with mental health professionals.

Integrated Mental Health Care in the community is undertaken via the Response, Early Intervention and Assessment in the following program, Community Mental Health program, Early Psychosis Intervention Program, Community Mental Health Team, and Community Psychogeriatric Program, which address the needs of different demographic and disease groups. The Mental Health–General Practitioner (GP) Partnership allows for GPs to receive training to manage patients with stable mental illnesses [103]. Patients with stable or chronic psychiatric illnesses may be managed in the community, which is more affordable and accessible. Moreover, the shift into the community setting is often less stigmatizing for the patient than follow-ups in a state mental institution [104].

Individual placement and support (IPS) programs are effective in aiding Singaporeans with psychiatric illnesses in obtaining and keeping competitive jobs [105]. IPS reduced stress after they found work and interactions with co-workers dispelled negative self-thoughts and self-stigma [105]. As part of the NMHB, a Job Club was established and run collaboratively by occupational therapists, medical social workers and job placement officers [101]. While helping the mentally ill to find suitable employment and reintegrate into society, the IPS programs also change employer perceptions and reduce stigma.

In general, Singaporeans who can correctly recognize a mental illness were less inclined to seek help from informal sources, while having an increased preference to seek help from mental health professionals. They also had less personal and perceived stigma [106]. Thus, improved general awareness and mental health literacy can be immensely beneficial to the broader community. Public education campaigns and portrayal in the mass media has led dementia (66.3% of respondents), alcohol abuse (57.1%), and depression (55.2%) to become the most well-recognized conditions [107]. However, conditions such as obsessive compulsive disorder and schizophrenia were only recognized by 28.7% and 11.5%, respectively of the respondents surveyed [107]. Younger age and higher educational status correlated with better recognition of psychiatric disorder [107].

Anti-stigma activities in Singapore are aimed at improving mental health literacy, increasing access to integrated mental health care in the community setting, and improving the employability and reintegration of the mentally ill into the community. As there is still a high level of stigma against mental illness in Singapore, the degree of success and the impact of these anti-stigma activities are potential areas for future research.

### 3.5. Stigma Related to Psychiatric Illnesses and Advancement of Anti-Stigma Activities in Korea

Korea is the fifth largest economy in Asia, and its population was estimated to be 50.8 million [108]. The lifetime prevalence rate for psychiatric illnesses in Korea was 27.6%, and the suicide rate has remained high, with 29.1 people out of every 100,000 have committed suicide [109]. In Korea, demographic factors including education, age, gender and marital status play an important role in influencing stigma and mental health service utilization. Previous researchers found that educational level had a significant positive relationship with the utilization of mental health services [110]. Highly educated Koreans are more likely to recognize their psychiatric illnesses, obtain information about mental illness and access to mental health services. In addition, studies have also shown that highly educated Koreans encounter less stigma related to psychiatric illness, resulting in higher rates of utilization [110].

Besides education, age plays an important role in mental health utilization. Jang et al. (2018) found that individuals above 70 years old were less likely to receive mental health consultation as compared to the 19–29 years old group [110]. Perceived stigma on the use of mental health services still affects older Koreans. Traditional Confucian values consider mental disorders as internal problems to be tolerated but which cannot be treated. This value caused older Koreans to avoid mental health services due to its association with personal weakness [111]. In addition, older Korean men with less education had a more negative perception and stigma against suicide and depression respectively [112]. Gender also plays a key role in affecting mental health-seeking behavior. In Korea, women were also shown to have a higher rate of mental health consultations compared to men [113,114]. This difference might be attributed to the higher perceived stigma by men as compared to women [115]. In terms of marital status, divorced Koreans received more psychiatric consultations when compared to married counterparts [113].

For psychiatric patients who do not seek treatment, the most common reasons included the wish to handle illnesses on their own, denial of psychiatric diagnosis, and spontaneous recovery [116]. The above reasons could originate from the passive-avoidance coping strategy against stigma, which is commonly adopted by Koreans [113]. Koreans with alcohol dependence were four times less likely to seek treatment as compared to alcohol-dependent Americans, although there was a significantly higher prevalence of alcohol dependence in Korea (5.1%) when compared to the United States (4.4%) [117]. This observation was caused by the presence of stigma and negative views from the society towards Koreans with substance use disorders or mental health problems, resulting in Koreans being less likely to seek mental health treatment due to embarrassment.

Besides public stigma against mental illness, internalized stigma has also been shown to facilitate the relationship between self-perceived cognitive deficits and quality of life among Koreans suffering from schizophrenia [118]. More importantly, self-stigma has also been associated with increased suicidal ideation and attempts [119]. Among Korean psychiatric patients, low self-esteem, high levels of insight, hopelessness, and social conflict are independent predictors of internalized stigma [114]. Hence, it would be of utmost importance to target self-esteem, hopelessness, and social coping skills in addition to psychoeducation to reduce internalized stigma [114].

The discrimination experienced by psychiatric patients in Korea includes emotional shunning, difficulty in purchasing medical insurance, and detrimental influences on their reputation and career [120]. In some professions such as firefighters who suffer from post-traumatic stress disorder, stigma is a barrier to seek psychiatric treatment [121]. Similarly, Koreas workers who suffer from depression were found to be less likely to use mental health services than unemployed individuals [115]. Undetected or untreated depression as the most important factor leading to suicide, thus emphasizing the importance to decrease stigma by removing the barrier of seeking treatment, and to raise awareness of the importance of psychiatric treatment [122] In Korea, the early version of the Mental Health Act included people with mild mental illness requiring only outpatient treatment as ‘mentally ill persons’ [123]. This legislation might prevent employed individuals from using mental health services due to fear of being labeled as mentally ill. In 1995, the Mental Health Act was revised to put forward the fundamental principle of respect towards mentally ill persons and shifted focus to community-based mental health services [109].

The stigma surrounding Koreans with mental illness not only affects themselves but their family members which is known as ‘family stigma’. Besides avoiding social interactions to conceal the fact that a patient suffers from a psychiatric illness, the occupation and interpersonal relationships of other family members might be affected [124]. Stigma has a negative impact on self-esteem and forces family members preventing a relative with psychiatric illness to seek treatment [125]. In the community, it has been shown that Koreans with psychiatric disorders residing in a neighborhood with a strong sense of mutual support reported lesser perceived stigma [126]. In contrast, a neighborhood with a large number of psychiatric patients with disabilities reported higher levels of perceived and experienced stigma. The positive association between stigma and psychiatric disorders in neighborhood emphasizes the importance of community engagement strategies to reduce public stigma [126]. The Gyeonggi Mental Health Commission has been providing services for Koreans with psychiatric illness and initiated cultural activities related to mental health, with the possibility that culture can increase the public’s understanding of psychiatric illnesses and lead to the process of recovery for persons with psychiatric illnesses [127]. Programs such as ‘safeTALK’, in partnership with the Korea Association for Suicide Protection, is another example of a community program that focuses on training the youth to understand about mental health and aims to change the public perception and awareness of suicide as a preventable health issue [128].

In 2000, an article calling for a survey to be conducted every five years to evaluate the status of mentally ill persons was added into the Mental Health Act. In 2008, an article was added to call for the formulation of the national mental health project plan every five years [123]. In 2016, the Act was revised to include involuntary hospitalization procedures allowing for hospitalizations for up to six months. This revision raised concerns about increasing stigma [129]. To address this concern, the Act on the Improvement of Mental Health and the Support of Welfare Services for Mental Patients was introduced in 2017. This new Act allows psychiatric patients to receive treatment in the community for their autonomy [123]. These revisions of the law will lay the foundations on which future anti-stigma programs.

The Korean Government has launched various initiatives, including nationwide public education [127], the National Mental Health Five-Year Plan [109], and the National Strategy for Suicide Prevention [113] to change the perception of mental illness and reduce the stigma. In addition, the National Center for Mental Health was established with a mission of “Happiness through Better Mental Health”. The organization consists of a Division of Mental Health Services to focus on performing public mental health services, education and training. The division also provides mental health services in schools and workplaces [130].

The Korean Neuropsychiatric Association and Korean Society of Schizophrenia Research have also changed the term “schizophrenia (Jungshinbunyeolbyung)” to “attunement disorder (Johyeonhyung)” in 2011. The term “schizophrenia” is a stigmatizing term and causes misconceptions that the condition is untreatable and patients having a dysfunctional personality [131]. Thus, renaming schizophrenia with a reformulation of the concept of the disease implies that “re-tuning” of minds is expected to reduce stigma and discrimination associated with the term schizophrenia [132]. A study assessing the attitudes towards this renaming was conducted and showed a significant positive change in perception among mental health practitioners. University students found that the term “attunement disorder” was a less severe disorder with higher treatability as compared to “schizophrenia” [133].

### 3.6. Stigma Related to Psychiatric Illnesses and Advancement of Anti-Stigma Activities in Thailand

Since King Chulalongkorn established the first psychiatric institute in Thailand, the year of 2019 marks the 130th anniversary of the application of Western-style mental illness care in Thailand. Over this period, the diagnosis and treatment of mental diseases have experienced rapid advancements. Simultaneously, much research on psychiatry from Thailand has been conducted and published in international journals. Surprisingly, research on anti-stigma activities related to mental illnesses is lacking, and no articles on this topic were published in international journals until 2019. Of the 40 published studies following the PRISMA guidelines, only three studies were relevant to psychiatric stigma. However, two of these studies focused on validation of a stigma scale. Only one was a qualitative study was conducted by psychiatric nurses, which concluded that the stigma faced by patients with schizophrenia was caused by the patients’ perceived barriers that exist in the health care services of Thailand [134].

This knowledge gap may highlight the severity of the problem of stigma among psychiatric patients in Thailand, which is seriously underestimated and often ignored. Recent work on stigma related to psychiatric disorders has been published in Thai which is related to social sciences. This study involves the discourse analysis, a research method for studying written or spoken language with its social context of mental illnesses [135].

One of the studies published in 2018 explored the discourse toward psychiatric illnesses represented in 19 novels by a famous Thai writer [136]. The writer attempts to introduce and educate readers on mental disorders. However, these novels, employing various literary techniques, suggest that psychiatric problems are a result of one’s inappropriate behaviors, which may enhance the level of stigma even further. A review article suggested that the root of stigma against mental disorders lied in the discrepancy that exists among three dominant concepts fighting for the approval of the general public, the traditional Thai view, and the Western view [135].

## 4. Discussion

This systematic review provided an overview of stigma related to mental illness and interventions to reduce stigma in China, Hong Kong, Japan, Korea, Singapore, and Thailand. There were similarities between an earlier review performed by Lauber and Rossler (2007) and our systematic review of stigma in Asian societies. Firstly, Asians with mental illnesses were considered as dangerous and aggressive, especially patients suffering from schizophrenia and bipolar disorder; second, psychiatric illnesses were less socially acceptable and being viewed as personal weakness; and third, stigma experienced by family members was pervasive and this is known as family stigma. Nevertheless, there were differences between the current review and previous review. First, this systemic review reported more initiatives to handle stigma in Asian societies than a decade ago; second, our review revealed initiatives to treat psychiatric patients in the community; and third, the role of supernatural and religious approaches to psychiatric illness was not prevailing in this systematic review. Table 1 summarizes existing policies against stigma and recommendations for future policy in six Asian societies.

This systematic review discovered new findings. Stigma toward people with mental illnesses exists to a substantial extent in Asian societies. In China, there was widespread discrimination against psychiatric patients by family members, nurses, and students. There were concerns that mental health professionals held discriminatory attitudes towards people with mental illnesses in China. Patients with a shorter duration of psychiatric illnesses experienced higher levels of stigma. In some Asian societies like Korea, highly educated people and women encounter less stigma related to psychiatric illnesses, resulting in higher rate of mental health service utilization. Similarly, communities with strong sense of mutual support reported less perceived stigma. The impacts of stigma include emotional shunning, difficulty in purchasing medical insurance, detrimental influences on reputations and career, prevention of help-seeking behaviors, leading to suicide attempts and difficulty to form therapeutic alliance. This systematic review also confirms that stigma and discrimination contribute to the treatment gap as reported in previous studies [7] and reduction of employment opportunities.

This systematic review highlighted other important findings. Media portraited negative themes and images of mental illnesses by emphasizing abnormal behavior, aggression, and danger. This bias was caused by a lack of knowledge to understand the nature of psychiatric illnesses by media and reporters. In Asian societies, mental hospitals or institutes were preserved to provide inpatient services for psychiatric patients, and the “institutional effect” is associated with additional stigma. Nevertheless, some Asian societies have shifted policy from an institutional based model of care to community-based approach. Traditionally, the prejudice against people with mental illnesses was influenced by cultural beliefs regarding psychiatric patients were weak but not sick and dangerous. Due to the recent effort of public education to enhance mental health literacy in Asia, psychiatric illnesses that are more prevalent and have higher chance to affect ordinary people such as dementia and depression are better recognized nowadays.

It is important to consider similarities and differences between Asian societies included in this systematic review and Western countries. There are similarities in predisposing factors for causing negative attitudes towards mental illnesses between some of the Asian and European societies. Negative attitudes towards mental illnesses were associated with male gender, older age, lower educational level, and living alone in Korea and Hungary [137]. Furthermore, Europeans with schizophrenia experience greater self-stigma than Europeans with depression [138,139], which is similar to findings of this review. In contrast to Western countries, there is less development and research in social capital in Asian societies. Social capitals include resources, social reciprocity, and trust in one’s social network [140]. Social capital was found to be a predictor of empowerment among Europeans with major depressive disorder and mediated effect of self-stigma [141]. Asian immigrants often experience depression and stigma in western countries [142,143]. Although migrant workers often experience depression and health inequality in Asian societies [144,145], this topic is not well studied in Asia.

The levels of stigma vary among different psychiatric disorders in Asian societies. For instance, schizophrenia and bipolar disorder are highly stigmatizing, and people with psychosis in Hong Kong are more likely to be perceived as violent and unpredictable as compared to people with other mental health problems. This bias can lead to high levels of experienced and anticipated discrimination in health care settings in Hong Kong. In Korea, older population with less education had higher prejudice against people who have mental illness and ignorance about psychiatric treatment. In Korea, passive avoidance coping strategy and embarrassment resulted in refusal to seek psychiatric treatment. Moreover, substance abuse is consistently associated with high rates of stigma and embarrassment in Korea that may discourage individuals with substance abuse problems from receiving psychiatry care.

In Asia, there are separate national programs to reduce stigma and discrimination. Reducing experienced and anticipated stigma among service users facilitates help-seeking and engagement with mental health care. For example, China launched the National Mental Health Work plan that requires the media to publicize correct information about mental health. In Singapore, there is a similar and related program called National Mental Health Blueprint to promote primary prevention, improving the coordination and enhancing quality of psychiatric services. Similar programs are also running in Korea (Like Community Engagement Strategies, safeTALK to engage youth for suicide prevention; Happiness through Better Mental Health). Nevertheless, there was no data available regarding any increase in access to mental health care of the above programs. In the future, anti-stigma programs in Asian societies can be better organized and target at specific groups at different levels in the community. A good example is a large scale project called Optimizing Suicide Prevention Programs and their Implementation in Europe (OSPI-Europe) which targeted at four specific levels: The first level aimed to increase the population’s knowledge about a psychiatric condition (e.g., depression) and its treatment as well as to decrease stigmatizing attitudes by means of a public media campaign. The second level involved training primary care physicians. The third level aimed at training community facilitators. The fourth level involved supporting patients and their relatives [146]. These initiatives will enhance acceptance of treatment offered by mental health professionals.

Another strategy to reduce stigma is to rename some of the psychiatric illnesses. The term schizophrenia was modified in Hong Kong (from a split in mind to incoordination in thoughts and perception) and Japan (from mind splitting disease to integration disorder). As a result, attitudes toward people with schizophrenia have become more acceptable in these Asian societies, which are different from the situation in Germany where attitudes towards people with schizophrenia have worsened [147]. Furthermore, media and reporters need to adopt a neutral stance when reporting news related to psychiatric patients.

Anti-stigma programs can incorporate new initiatives aimed at multiple modalities (e.g., lectures, role-plays, videos, educational materials, nationwide psychoeducation targeted at insight and self-esteem, employment placement, or job club) and at the general public (e.g., invitation of the general public to visit psychiatric hospitals and perform voluntary work, mental health first aid courses), and operates at multiple levels (i.e., national social marketing campaigns and regional activities, school-based education programmes, partnership with GP).

Stigma and discrimination can impede access to mental health services. Several Asian countries amended the legislation and Mental Health Law. In China, the revised Mental Health Law stipulated the legitimate rights and interests and confidentiality of psychiatric patients. Anti-discrimination law was passed to prohibit discrimination, insult, and maltreatment of psychiatric patients. In Korea, the Mental Health Act was revised to emphasize on the respect towards psychiatric patients, increased availability of psychiatric services in the community and formulate national mental health project every five years. In the future, Asian societies need to build a supportive environment to involve family members, employers, and allied health professionals who will reduce the negative effects of psychiatric illness-related stigma and consequently build self-esteem and optimism of psychiatric patients [141].

Based on the findings of this systematic review, we propose four research directions in Asian societies. First, more Asian societies need to assess the personal and perceived stigma of mental illnesses. Personal stigma is defined as an individual’s personal beliefs and thoughts about a psychiatric condition [146], while perceived stigma represents an individual’s perception of what other people from the same society think and feel about a psychiatric condition [148]. Second, future research will assess Asians’ views on biological explanations of mental illnesses. Third, Asian societies can consider developing large scale anti-stigma programs like OSPI-Europe and monitor the potential interaction between different Asian cultures and psychiatric illnesses on stigma and help-seeking behaviour in future decades. Fourth, further research will examine the impact of change in mental health legislation in some Asian societies. Finally, the fact that this systematic review found very few studies from Thailand on anti-stigma activities employed in the psychiatric population is a strong indicator of the urgent need for research in some Asian societies. National data regarding stigmatization related to mental illnesses are urgently needed in order to develop effective anti-stigma activities to achieve a concrete improvement in quality of life of psychiatric patients in Asian societies.

The main limitation of this systematic review is that the findings were based on six Asian societies only. These six countries have the sociocultural background and economic developments that are different from other Asian countries. When applying the above strategies to other Asian countries, modification is required.

## 5. Conclusions

This review provides an overview of the available scientific evidence that points to three areas of needed intervention to reduce and ultimately eliminate stigma related to psychiatric illnesses in Asian societies. First, comprehensive efforts are needed to create and maintain opportunities that facilitate health and its determinants at the level of the local community. Second, health care providers and institutions should give greater emphasis to prevention, address patients’ social risk factors and needs, and to ensure that every client receives appropriate and high-quality psychiatric care. Third, major new initiatives are needed to inform the public and policymakers about the nature and extent of inequities in mental health; to enhance individual and community capacity and build public empathy and political will to effectively address them. Fourth, Asian countries also differ from each other in several socio-cultural dimensions. Thus, it is important to understand their differences and apply a culturally sensitive approach to handle stigma related to psychiatric illnesses in Asia.

## Figures and Tables

**Table 1:** Summary of existing policies against stigma and recommendations for future policy in six Asian societies.

Name of Asian Society	Summary of Existing Policies against Stigma	Recommendations for Future Policies
China	The National Mental Health Work Plan destigmatizes psychiatric illnesses and informs the public that psychiatric illnesses are treatable. National legislation and public education call for more respect towards psychiatric patients among the general public. Mental health law stipulates the legitimate rights and interests of psychiatric patients.	Anti-discrimination law is required to effectively protect the legitimate rights and interests of psychiatric patients in China Anti-stigma campaign for caregivers of psychiatric patients Fair treatment of psychiatric patients at work Invite the public to be volunteers at psychiatric facilities Media need to adopt a natural stance when reporting psychiatric patients Psychoeducation for mental illnesses in people living in rural areas, people with low education background and journalists Reduction of stigma among psychiatric nurses
Hong Kong	Lectures, role-plays, videos, and educational materials were used to improve mental health literacy. Replacement of the old Chinese translation of schizophrenia as ‘mental split-mind disorder’ by a more objective name ‘dysregulation of thoughts and perception.’	A culturally sensitive approach to focus on Chinese belief of mental illnesses More anti-stigma work should focus on patients suffering from psychosis and schizophrenia
Japan	Renaming the old name of schizophrenia by a new name called “integration disorder.” Educational programs and social contact were found to reduce stigma.	Anti-stigma activities target at other major psychiatric illnesses (e.g., depressive disorder, bipolar disorder) Address problems associated with institutionalism. Increase of accessibility of psychiatric services to adolescents Reduction of stigma and enhancement of mental health service utilization Support building of psychiatric facilities in the communities
Singapore	The National Mental Health Blueprint promotes primary prevention, improving the coordination of psychiatric services, developing mental health professionals, enhancing mental health monitoring and the quality of psychiatric services in Singapore. The Health Promotion Board is the main driver for mental health promotion. Integrated Mental Health Care and partnership with general practitioners in the community. Individual employment placement and support programme to assist psychiatric patients in applying for competitive jobs.	Adopt a culturally sensitive approach to Chinese, Indian and Malay Singaporean Psychoeducation on mental illnesses should target the older population with a lower education background. Interventions should target at self-stigma of psychiatric patients Provide more inpatient beds for non-psychotic psychiatric patients outside the main psychiatric hospital. Policy to make it mandatory for insurance companies to cover psychiatric illnesses and not to exclude psychiatric patients from purchasing medical insurances.
Korea	Renaming schizophrenia with a reformulation of the concept of the disease implies that “re-tuning” of minds. Revised Mental Health Act put forward the fundamental principle of respect towards psychiatric patients and shifted focus to community-based mental health services. Government initiatives include nationwide public education, the National Mental Health Five-Year Plan and the National Strategy for Suicide Prevention.	Anti-stigma activities should target at dual diagnosis of substance abuse and psychiatric illnesses as well as internalized stigma and stigma experienced by family members A culturally sensitive approach to focus on the Confucian view of mental illnesses Policy to make it mandatory for insurance companies to cover psychiatric illnesses and not to exclude psychiatric patients from purchasing medical insurances. Psychoeducation on mental illnesses should target the older population with a lower education background.
Thailand	Policy against stigma related to psychiatric illnesses is still lacking.	Anti-stigma activities should target at the root of stigma, which lied in the discrepancy that exists among three dominant concepts fighting for the approval of the general public, the traditional Thai view, and the western view. More research is required to study anti-stigma activities related to psychiatric illnesses.
